# Sedentary Patterns and Systemic Inflammation: Sex-Specific Links in Older Adults

**DOI:** 10.3389/fphys.2021.625950

**Published:** 2021-02-05

**Authors:** Oscar Bergens, Andreas Nilsson, Konstantinos-Georgios Papaioannou, Fawzi Kadi

**Affiliations:** School of Health Sciences, Örebro University, Örebro, Sweden

**Keywords:** aging, physical activity, sedentary behaviors, inflammatory biomarkers, metabolic health

## Abstract

The study aimed to examine sex-specific associations between objectively measured sedentary patterns and pro- and anti-inflammatory biomarkers in older adults when considering the moderating impact of physical activity (PA). Accelerometer-based monitoring of sedentary patterns and PA was conducted in a population of older men (*n* = 83; age: 67.4 ± 1.5; height: 178.7 ± 6.6 cm; weight: 80.9 ± 10.6 kg) and women (*n* = 146; age: 67.4 ± 1.6; height: 164.2 ± 6.1 cm; weight: 64.6 ± 10.1 kg) aged 65–70. Blood samples were collected for the assessment of the inflammatory biomarkers C-reactive protein (CRP), fibrinogen, interleukin-6 (IL-6), IL-10, IL-18, and monocyte chemoattractant protein-1 (MCP-1). Data were analyzed using multiple linear regression models. Total and bouts of ≥10 min of sedentary time were inversely associated with the anti-inflammatory marker IL-10 in older men (accumulated sedentary time: β = −0.116; bouts: β = −0.099; all *p* < 0.05). Associations were independent of moderate-to-vigorous physical activity (MVPA) and total PA volume. In women, total and bouts of ≥10 min of sedentary time were detrimentally associated with the pro-inflammatory marker fibrinogen (accumulated sedentary time: β = −0.130; bouts: β = −0.085; all *p* < 0.05). Associations remained between accumulated sedentary time and fibrinogen when adjusting for MVPA and total PA volume. This study highlights sex-specific routes by which sedentary patterns impact on pro- and anti-inflammatory biomarkers in older adults. The findings support efforts to promote accumulation of time spent in PA at the expense of time in sedentary pursuits on low-grade inflammation in older men and women.

## Introduction

Leading a sedentary lifestyle is implicated with increased risk of several chronic conditions, including cardiovascular and metabolic diseases (Dunstan et al., 2011). In this respect, excessive amounts of time in a sedentary state have been linked to obesity, hypertension, dyslipidemia, and hyperglycemia, collectively known as the metabolic syndrome (Edwardson et al., 2012). While the pathophysiological mechanisms underlying progression of metabolic disorders are not fully clarified, alterations in the systemic inflammatory environment, have been highlighted as an important mediator (Franceschi and Campisi, 2014). Elevations in circulating levels of several pro-inflammatory mediators by advancing age promote as state of low-grade systemic inflammation (inflammaging). The two hepatocyte-derived acute-phase proteins; C-reactive protein (CRP) and fibrinogen, together with the monocyte chemoattractant protein (MCP)-1, a member of the C-C chemokine family and a potent chemotactic factor for monocytes, and the cytokines interleukin-6 (IL-6) and IL-18, represent biomarkers contributing to a pro-inflammatory systemic environment (Stec et al., 2000; Koenig et al., 2004; Zirlik et al., 2007; Jung and Choi, 2014). Interestingly, systemic inflammation in older adults is further influenced by a declined level of IL-10 (Meador et al., 2008), an anti-inflammatory cytokine mainly produced by macrophages and responsible for suppressing inflammatory responses (Asadullah et al., 2003; Dorneles et al., 2016).

Associations have been evidenced between daily sitting time, including TV viewing time, and the two common acute-phase reactants CRP and fibrinogen, indicating a potential detrimental influence of a sedentary lifestyle on systemic inflammation (Rudnicka et al., 2011; Yates et al., 2012; Gennuso et al., 2013; Henson et al., 2013; Hamer et al., 2015; Howard et al., 2015). However, previous studies have shed light on sex-specific differences in levels of pro- and anti-inflammatory biomarkers, where CRP and fibrinogen levels have been found to be significantly higher in women than men (Cartier et al., 2009; Rudnicka et al., 2011; Yates et al., 2012; Howard et al., 2015). Further, stronger relationships between inflammation and sedentary behavior have been indicated in women compared to men (Pereira et al., 2012; Yates et al., 2012; Howard et al., 2015). It is currently proposed that genetic, hormonal, and environmental factors represent underlying mediators behind sex-differences in the production of cytokines and chemokines by innate immune cells (Klein and Flanagan, 2016). For example, following menopause women experience a significant reduction in estrogen levels, which is associated with adverse inflammatory events (Campesi et al., 2016; Razmjou et al., 2016). Therefore, it may be hypothesized that sex-specific links between sedentary lifestyle and different pro- and anti-inflammatory factors exist and need to be further clarified. Furthermore, systemic inflammation is generally assessed by a few common inflammatory biomarkers (typically CRP, fibrinogen, and IL-6), which does not necessarily reflect the full spectrum of the inflammatory process in older adults. We have previously reported adverse associations between single and composite inflammatory score with adiposity in older adults suggesting at the importance of investigating the implications of several cytokines in regard to the inflammatory load in older adults (Bergens et al., 2019). Therefore, a better understanding of the relationship between sedentary behaviors and systemic inflammation would require investigation of inflammatory biomarkers encompassing clinically established ones, as well as novel inflammatory mediators. Importantly, when investigating these links attention should be paid to the pattern of sedentary time accumulation over the day, i.e., whether characterized by uninterrupted prolonged time periods or by shorter periods with frequent breaks in sedentary time. This includes analysis of different time bout lengths and number of breaks in sedentary time.

Another important question to address is whether potential links between sedentary patterns and pro- and anti-inflammatory biomarkers are independent of physical activity level. Some studies have previously reported independent associations between sedentary time and a few clinical markers of inflammation, indicating that moderate-to-vigorous physical activity (MVPA) time may not mitigate the detrimental impact of sedentary time (Healy et al., 2011b; Yates et al., 2012; Gennuso et al., 2013; Hamer et al., 2015; Howard et al., 2015). However, as time in MVPA represents a small part of the daily time, residual confounding from remaining time spent in lower intensity physical activity (PA) may still be present. Therefore, daily PA volume, incorporating both low and high intensity PA, should be considered in new investigations aiming to clarify the unique impact of sedentariness on systemic inflammation in older adults. Moreover, given the potential impact of dietary habits on inflammatory profiles, indicated in previous research (Nilsson et al., 2019), risk of confounding by diet when elucidating links between sedentariness and inflammation needs to be addressed.

Taken together, to what extent sedentary patterns are associated to systemic inflammation independent of PA level in older adults, and whether these links are sex-specific, remains to be clarified. Such knowledge is warranted in order to tailor sex-specific guidelines on health-related PA behaviors in order to dampen age-related systemic inflammation. Therefore, the aim of the present study was to explore relationships between objectively assessed sedentary patterns and pro- and anti-inflammatory biomarkers in older men and women when considering potential moderation by PA level.

## Materials and Methods

### Participants

The present study included 252 community-dwelling older men and women, 65–70 years old, recruited within the frame of the EURODIET project. Inclusion criteria: age between 65 and 70 years. Exclusion criteria: overt disease such as diabetes mellitus or diagnosed coronary heart disease, disability with respect to mobility, smoking, and anti-inflammatory treatment. All clinical investigations were conducted in accordance with the Declaration of Helsinki and all participants provided written informed consent. The study was approved by the regional ethics committee of Uppsala, Sweden.

### Assessment of Physical Activity Behaviors

Accelerometer-based assessment of PA was monitored with the Actigraph GT3x activity monitor (Actigraph, Pensacola, FL, United States) worn around the waist for 7 days. Briefly, participants were instructed to wear the monitor during all waking hours (except water-based activities) and note non-wear time. Inclusion criteria were that the activity monitor was worn for a minimum of 4 days with at least 10 h of wear time per day. Non-wear time was defined as a minimum of 60 min of continuous zero counts. Sedentary wear time was defined as all <100 counts, and MVPA as >2,020 in accordance with previous research (Troiano et al., 2008; Lynch et al., 2011). Breaks in sedentary behavior were defined as any 60 s of ≥100 counts. Time spent in sedentary patterns was retrieved as three separate outcomes: total accumulated sedentary time, amount of time occurring in bouts of ≥10 and ≥30 min. Breaks in sedentary behavior were expressed in relation to occurrences per sedentary hour (Break rate). Total accelerometer counts were retrieved as a marker of total volume of PA.

### Assessment of Dietary Inflammatory Score

Information on dietary habits was collected using a validated food frequency questionnaire (Johansson et al., 2010). A dietary inflammatory index (DII) score was derived from 30 food items following calculation procedures presented elsewhere (Shivappa et al., 2014).

### Assessment of Laboratory Measurements and Metabolic Risk

Blood samples were collected by venipuncture between 8.00 and 10.00 am after an overnight fast. For this study, six inflammatory biomarkers previously linked to metabolic syndrome were selected based on previous research (Nilsson et al., 2018; Bergens et al., 2019). High-sensitivity CRP was measured using fully automated immunoturbidimetric assay. Fibrinogen was determined using an automated immunoassay method with a polyclonal rabbit anti-human antibody (Dako, Glostrup, Denmark). IL-6, IL-10, IL-18, and MCP-1 were analyzed using the Olink Proseek Multiplex Inflammation panel (Olink, Uppsala, Sweden). Briefly, a pair of oligonucleotide-labeled antibodies are pairwise bound to the target protein present in the sample and a new PCR target sequence is formed by a proximity dependent DNA polymerization event. The resulting sequence is subsequently detected and quantified using standard RT-PCR. All OLINK biomarkers had complete Normalized Protein eXpression (NPX) detectability (0% missing data frequency) and intra-assay variations were as follows: MCP-1 (6%), IL-6 (6%), IL-10 (7%), and IL-18 (6%).

Metabolic risk outcome variables included in the International Diabetes Federation (IDF) definition of the metabolic syndrome were assessed (Alberti et al., 2006). Waist circumference was measured to the nearest 0.1 cm with a steel tape at the midpoint between iliac crest and lower costal margin. Systolic and diastolic blood pressure were measured manually after a 10-min rest in the supine position. Fasting blood triglycerides and HDL-cholesterol were assessed on plasma samples using chemistry kits from Ortho-Clinical Diagnostics on a Vitros-5.1 analyzer platform (Clinical Diagnostics, Raritan, NJ, United States) and plasma blood glucose was assessed using the Reflotron Plus^®^ system (Roche Diagnostics Limited, Rotkreuz, Switzerland). A clustered metabolic risk score was created based on the assessment of measured metabolic risk outcome variables (level of triglycerides, HDL-cholesterol, glucose, waist circumference, and mean blood pressure). First, standardized values (*z*-scores) of each outcome variable were expressed. *Z*-scores on HDL-cholesterol were inverted before calculating the average *z*-score based on all standardized outcomes.

### Statistical Analysis

Data are presented as means ± SD. Differences between men and women were determined by independent sample *t*-test. To retrieve comparable effect outcomes from variables with different original units of measurement, all inflammatory biomarkers were standardized (*z*-score) before regression modeling. All variables were checked and transformed when necessary to fit a normal distribution. Prior to analysis, sedentary behaviors and MVPA were modeled in 30-min periods. All models were adjusted by accelerometer wear time. First, before adjusting for PA behaviors, linear associations between each inflammatory marker and time spent in sedentary behavior and break rate were assessed (Model 1). Second, linear regression models were conducted to determine the strength of associations between measured inflammatory biomarkers and sedentary behavior when adjusting for MVPA (Model 2) or total accelerometer counts (Model 3) and metabolic risk score (Model 4). All models were stratified by sex. Potential significant influences of age and dietary inflammatory score on inflammatory outcomes were first checked using simple linear regressions. As no significant associations were detected, age and dietary inflammatory score were omitted in final regression models in order to retain statistical power. Assumptions for regression models including linearity, homoscedasticity, and multicollinearity between independent variables were checked. Level of significance was set to *p* < 0.05. All statistical analyses were performed using SPSS version 26. *A priori* power calculation revealed a small to moderate effect size to be detected with a power of ≥80% given the current sample size and alpha set to 0.05.

## Results

A total of 229 community-dwelling older adults (146 women and 83 men) with complete data on all variables were included in the final analysis. Eight women and 15 men had either incomplete accelerometer recordings, missing biological data, were current smokers or used prescribed anti-inflammatory medication. On average, participants wore the activity monitor for 6.1 ± 0.9 days with 14 ± 1.2 h per day, without differences between the sexes. Data on body composition, CRP and fibrinogen and sedentary patterns are shown in Table 1. Women spent significantly less daily time in sedentary activities compared to men, both in terms of total accumulated time (*p* < 0.05) and in continuous time bouts of at least 10 min (*p* < 0.05) and 30 min (*p* < 0.05), respectively. Women also had significantly more breaks per sedentary hour (i.e., higher break rate) compared to their male counterparts (*p* < 0.05) (Table 1). While average PA level (women: 375.8 ± 136.9 counts per min; Men: 376.0 ± 111.8 counts per min) and time spent in MVPA (women: 42.3 ± 25.0 min; men: 40.9 ± 21.2 min) were similar between sexes, women spent significantly more time in LPA (women: 125.4 ± 51.1 min; men: 275.2 ± 69.6 min, *p* < 0.05). There were no significant differences between men and women in levels of the clinically well-established inflammatory biomarkers CRP and fibrinogen.

**TABLE 1:** Body composition (mean ± SD), levels of CRP and fibrinogen, and sedentary behavior variables in older men and women.

	**Women (*n* = 146)**	**Men (*n* = 83)**
Age (year)	67.4 ± 1.6	67.4 ± 1.5
Height (cm)	164.2 ± 6.1	178.7 ± 6.6*
Weight (kg)	64.6 ± 10.1	80.9 ± 10.6*
Body mass index (kg/m^2^)	24 ± 3.6	25.2 ± 3*
Waist circumference (cm)	80 ± 9.3	93.8 ± 9.6*
CRP (mg/L^–1^)^a^	1.1 ± 1.9	1.1 ± 2.0
Fibrinogen (g/L^–1^)	3.2 ± 0.6	3.2 ± 0.5
Total sedentary time (min)	494.0 ± 67.4	514.1 ± 72.1*
Bouts of ≥10 min sedentary time (min)	314.4 ± 70.1	350.4 ± 72.6*
Bouts of ≥30 min sedentary time (min)	125.4 ± 51.1	149.7 ± 55.7*
Break rate (n/h)	10.0 ± 1.9	8.9 ± 1.8*

^*a*^Presented as geometric mean ± SD. *p < 0.05 between men and women.

We sought to determine the influence of different sedentary behaviors on levels of inflammatory biomarkers. First, influences of daily amounts of sedentary time, either derived in uninterrupted time bouts of ≥10 or ≥30 min or accumulated over a day, were explored in sex-stratified models. In women, a higher amount of accumulated sedentary time was significantly related to higher levels of the pro-inflammatory biomarkers IL-6 (*p* < 0.05) and fibrinogen (*p* < 0.05) (Table 2). A positive relationship with fibrinogen was also evident when analyzing amount of sedentary time derived in continuous bouts of at least 10 min (*p* < 0.05) (Table 2). Besides links to sedentary time, an inverse significant relationship was further observed between break rate and fibrinogen level (*p* < 0.05). Importantly, all observed associations remained significant after further adjustment for time spent in MVPA. However, only the link between total accumulated sedentary time and fibrinogen remained after adjusting for total accelerometer counts instead of MVPA time (*p* < 0.05) (Table 2). Moreover, the detrimental impact of sedentary time on fibrinogen level remained after further adjustment by metabolic risk score (model 2: β = 0.105, 95% CI: 0.023–0.186; model 3: β = 0.099, 95% CI: 0.002–0.195, all *p* < 0.05). Finally, no relationships with other inflammatory biomarkers were observed (Table 2).

**TABLE 2:** Associations (β-coefficients, 95% CI) between dimensions of sedentary time and inflammatory biomarkers in older women.

	**Accumulated sedentary time**	**Bouts of ≥10 min sedentary time**	**Bouts of ≥30 min sedentary time**	**Break rate**
				
**Model 1**				
CRP^a^	0.071 (−0.010 to 0.152)	0.042 (−0.030 to 0.114)	−0.001 (−0.097 to 0.095)	−0.240 (−0.793 to 0.313)
Fibrinogen	0.130 (0.051–0.208)*	0.085 (0.015–0.155)*	0.069 (−0.025 to 0.163)	−**0.656 (**−**1.199 to**−0.112)*
MCP-1	−0.040 (−0.122 to 0.043)	−0.019 (−0.091 to 0.054)	−0.003 (−0.101 to 0.094)	0.117 (−0.434 to 0.668)
IL-6	0.101 (0.020–0.182)*	0.059 (−0.013 to 0.130)	0.060 (−0.036 to 0.157)	−0.310 (−0.858 to 0.239)
IL-10	−0.021 (−0.062 to 0.103)	0.031 (−0.042 to 0.103)	0.032 (−0.065 to 0.129)	−0.306 (−0.855 to 0.243)
IL-18	0.060 (−0.022 to 0.142)	0.039 (−0.033 to 0.112)	0.006 (−0.091 to 0.104)	−0.186 (−0.736 to 0.365)
**Model 2**				
IL-6	0.094 (0.009–0.178)*	–	–	–
Fibrinogen	0.125 (0.043–0.207)*	0.081 (0.010–0.151)*	–	−**0.660 (**−**1.201 to**−0.119)*
**Model 3**				
IL-6	0.078 (−0.022 to 0.178)	–	–	–
Fibrinogen	0.110 (0.011–0.208)*	0.058 (−0.019 to 0.135)	–	−0.479 (−0.1,031 to 0.072)

Model 1, adjusted for wear time; Model 2, model 1 + MVPA; Model 3, model 1 + total accelerometer counts. ^*a*^*n* = 144; *p < 0.05. Bold values indicates that to highlight statistically significant values.

In men, higher amounts of sedentary time derived either accumulated or in continuous bouts of at least 10 min, were significantly related to lower levels of the anti-inflammatory biomarker IL-10 (*p* < 0.05) (Table 3). Further, a significant positive relationship between IL-10 and break rate was observed (*p* < 0.05), indicating a beneficial impact of breaking up sedentary activities on levels of this anti-inflammatory agent. Interestingly, adjustment for PA either by time in MVPA or total accelerometer counts did not attenuate the significant influence of sedentary behavior on IL-10 level (Table 3). Importantly, we further adjusted our models by metabolic risk score, which left significant associations unchanged (accumulated sedentary time: model 2, β = −0.124, 95% CI: −0.241 to −0.007; model 3, β = −0.146, 95% CI: −0.272 to −0.020; bouts of ≥10 min: β = −0.101, 95% CI: −0.199 to −0.004; model 3, β = −0.110, 95% CI: −0.210 to −0.011; break rates: β = 0.140, 95% CI: 0.011–0.269; model 3, β = 0.139, 95% CI: 0.016–0.261; all *p* < 0.05). Notably, no other biomarkers of inflammation were significantly associated with sedentary behaviors in the male participants (Table 3).

**TABLE 3:** Associations (β-coefficients, 95% CI) between dimensions of sedentary time and inflammatory biomarkers in older men.

	**Accumulated sedentary time**	**Bouts of ≥10 min sedentary time**	**Bouts of ≥30 min sedentary time**	**Break rate**
				
**Model 1**				
CRP^a^	−0.001 (−0.118 to 0.116)	−0.021 (−0.118 to 0.076)	−0.031 (−0.151 to 0.089)	0.059 (−0.065 to 0.182)
Fibrinogen	−0.019 (−0.135 to 0.098)	−0.041 (−0.137 to 0.056)	−0.088 (−0.207 to 0.030)	0.032 (−0.092 to 0.156)
MCP-1^a^	0.037 (−0.078 to 0.153)	0.042 (−0.054 to 0.138)	0.046 (−0.072 to 0.165)	−0.042 (−0.054 to 0.138)
IL-6	0.007 (−0.111 to 0.125)	−0.030 (−0.130 to 0.069)	−0.055 (−0.175 to 0.066)	0.060 (−0.69 to 0.189)
IL-10	−**0.116 (**−**0.230 to**−0.002)*	−**0.099 (**−**0.194 to**−0.004)*	−0.113 (−0.230 to 0.005)	0.130 (0.010–0.251)*
IL-18	−0.061 (−0.177 to 0.055)	0.060 (−0.156 to 0.037)	−0.059 (−0.179 to 0.060)	0.050 (−0.073 to 0.174)
**Model 2**				
IL-10	−**0.115 (**−**0.230 to**−0.001)*	−**0.099 (**−**0.194 to**−0.003)*	–	0.134 (0.007–0.261)*
**Model 3**				
IL-10	−**0.136 (**−**0.261 to**−0.011)*	−**0.105 (**−**0.204 to**−0.007)*	–	0.132 (0.010–0.254)*

Model 1, Adjusted for wear time; Model 2, model 1 + MVPA; Model 3, model 1 + total accelerometer counts. ^*a*^*n* = 82; *p < 0.05. Bold values indicates that to highlight statistically significant values.

## Discussion

The present study revealed sex-specific links between sedentary time patterns and biomarkers of low-grade systemic inflammation. Higher accumulated amounts of sedentary time were related to higher levels of the pro-inflammatory biomarkers IL-6 and fibrinogen in women, whereas an inverse relationship with the anti-inflammatory biomarker IL-10 was observed in men. Interestingly, time spent in MVPA did not offset the detrimental impacts of accumulating excessive amounts of sedentary time in both sexes. However, adjustment for total PA volume attenuated observed links with IL-6 in women.

It has been hypothesized that biological sex interacts with regulation of systemic inflammation, which should be considered when elucidating impacts of sedentariness on inflammatory status. Here we show detrimental sex-specific links for levels of IL-6, fibrinogen, and IL-10, which were generally confirmed by the association between a higher break rate and a more beneficial inflammatory profile. These findings are in accordance with previous observations highlighting stronger associations between sedentary time and levels of fibrinogen and IL-6 in women compared to men (Pereira et al., 2012; Yates et al., 2012; Howard et al., 2015). This is also in line with previous clinical and experimental findings showing compelling evidence of marked sex differences in inflammatory responses, where women exhibit a more pronounced pro-inflammatory response during acute infections (Van Eijk et al., 2007; Klein and Flanagan, 2016). For example, women show significantly higher increases in the pro-inflammatory biomarkers TNF-α and IL-6 compared to men, whereas the anti-inflammatory cytokine IL-10 was significantly higher in men (Engler et al., 2016). The significant difference in inflammatory response was in young men and women after administrating a low-dose bacterial endotoxin. It was observed that women mounted a stronger pro-inflammatory response to the endotoxin, while IL-10 was significantly raised in men. It is currently proposed that differences in hormonal levels, including estrogen, partly explain sex-specific modulations of the pro- and anti-inflammatory systemic environment (Rettew et al., 2009). Altogether, these findings suggest that biological sex needs to be considered when depicting factors involved in the regulation of systemic inflammatory environment and its relationship to PA behaviors in older adults.

An important finding of the present study was that total daily PA rather than time in MVPA attenuates links between sedentary patterns and inflammation in older women. Generally, time spent in MVPA has been considered when exploring the impact of sedentary patterns on systemic inflammation (Allison et al., 2012; Gennuso et al., 2013; Hamer et al., 2015; Howard et al., 2016; Parsons et al., 2017). For instance, Gennuso et al. (2013) observed that sufficient time spent in MVPA (≥ 150 min/week) derived from accelerometers did not significantly attenuate associations between sedentary time and CRP. However, as time in MVPA represents a small part of the daily time, residual confounding from remaining time spent in lower intensity PA may still be present. Thus, our findings are in favor of the hypothesis that detrimental links between sedentary patterns and systemic inflammation are partly explained by lower amounts of daily PA rather than by prolonged sedentariness itself.

Another important finding was the independent inverse relationship between sedentary patterns and IL-10 levels in men. This is in line with previous findings showing higher levels of IL-10 gene expression in active compared to sedentary older men (Ferrer et al., 2018). While assessment of activity status was achieved through questionnaires, Ferrer et al. (2018) demonstrated significant anthropometric differences between active and sedentary older adults and suggests that elevations in IL-10 is associated with a reduction in adiposity by PA. Given that IL-10 is a potent anti-inflammatory factor, this finding proposes that reducing sedentary behaviors could promote anti-inflammatory processes in older men across different levels of PA and metabolic risk. Although exact mechanisms underpinning the regulation of IL-10 levels remain unclear, sedentary behaviors characterized by low muscle activity may contribute to lower levels of IL-10 and their main cellular source (Treg cells) (Wang et al., 2012; Dorneles et al., 2016).

Interestingly, unlike previous studies, we did not observe a significant association between sedentary behavior and the pro-inflammatory biomarkers CRP, IL-18, and MCP-1 (Healy et al., 2011b; Pereira et al., 2012; Howard et al., 2015). There are several possible reasons for the discrepant findings. Firstly, there are differences in age, with Healy et al. (2011b) and Howard et al. (2015) also including younger and middle-aged adults, and Pereira et al. (2012) including only middle-aged adults. Secondly, time spent in sedentary behavior was assessed by interview-administered questionnaires of overall sitting behavior (Howard et al., 2015) or questionnaires on television-viewing and work-sitting (Pereira et al., 2012). Notably, CRP was significantly related to time in MVPA in both sexes, confirming previous observations that PA above light intensity is required to influence on this clinical marker of inflammation (Nilsson et al., 2018). The lack of associations with sedentary time denotes the complex inter-relationships between systemic inflammation and PA behaviors, where different dimensions of PA behaviors, including total amounts and time spent in different intensities, may have separate influences on inflammatory biomarkers depending on their pro- and anti-inflammatory actions.

Although sedentary behaviors lasting at least 30 min or longer are thought to be particularly detrimental to health, most of previous research supporting this hypothesis have been based on self-report measures, which is limited to cruder estimations of sedentary behavior compared to objective methods. Based on our objective assessment of sedentary patterns, significant associations were reported when analyzing time bouts lasting at least 10 min, while no corresponding links were observed for the 30-min bouts. A previous study investigating how to best capture prolonged sedentary behaviors when using accelerometers, including both ≥10- and ≥30-min bouts, concluded the ≥10-min bout to be an appropriate threshold to capture the prolonged nature of sedentary behavior and its relation to health outcomes (Kim et al., 2015). Thus, the contrasting result depending on whether a 10- or 30-min time window is employed, is likely due to the natural occurrence of sporadic breaks (i.e., ≥100 counts) when extending the time bout from ≥10- to ≥30-min, which would prevent detection of prolonged sedentary behaviors. Noteworthy, those who break-up their sedentary behavior more frequently (i.e., higher break rate) showed a more favorable inflammatory profile than those with a lower break rate, which highlights the beneficial health impact of avoiding prolonged sedentary behaviors. Interestingly, our results further indicate that the amount of daily sedentary time, regardless if occurring in uninterrupted time bouts or simply accumulated throughout the day, has a detrimental association to systemic inflammation. This further emphasizes the health-related potential of reducing daily amounts of sedentary time in favor of increasing PA level.

### Strength and Limitations

While our cross-sectional analysis highlights important associations between sedentariness and systemic inflammation, direction of the relationships cannot be determined. The older men and women included in this study may not be representative of broader populations of older adults, including differences in ethnicity and health status. Nonetheless, reported time spent in sedentary behavior are similar to what others have reported in older adults (Hagströmer et al., 2010). Further, PA behaviors were objectively assessed by accelerometers, which allowed capturing ambulatory activities but not postural data. However, the Actigraph activity monitor has been shown to accurately capture more than 80% of sedentary activities in a sitting or lying position (Carr and Mahar, 2012) and is frequently used to assess sedentary behaviors (Healy et al., 2011a).

Although our study revealed sedentary pattern-related impacts on different pro- and anti-inflammatory biomarkers, the systemic inflammatory environment is likely influenced by additional mediators not included in the present work. Further research is warranted to uncover molecular mechanism underpinning the systemic inflammatory profiles in aging populations.

### Conclusion

In conclusion, our findings confirm the hypothesis of sex-specific routes by which sedentary patterns impact on pro- and anti-inflammatory biomarkers in older adults. The detrimental impact of sedentary patterns is partly explained by total amounts of daily PA rather than by sedentariness itself. These findings hold clinical and public health implications, by reinforcing efforts aiming to promote daily PA at the expense of less sedentary pursuits in older men and women. Future efforts should be put on elucidating molecular mechanisms underlying observed links revealed in this study.

## Data Availability Statement

The original contributions presented in the study are included in the article/supplementary material, further inquiries can be directed to the corresponding author/s.

## Ethics Statement

The studies involving human participants were reviewed and approved by Regional ethics committee of Uppsala, Sweden. The patients/participants provided their written informed consent to participate in this study.

## Author Contributions

OB and AN: conception of the work, acquisition, analysis, and interpretation of data, drafting and revision of the work, and approval of final draft including agreement for accountability. FK: conception of the work, acquisition, analysis, and interpretation of data, revising and approval of final draft including agreement for accountability K-GP: acquisition of data, revision of manuscript, approval of final draft including agreement for accountability. All authors contributed to the article and approved the submitted version.

## Conflict of Interest

The authors declare that the research was conducted in the absence of any commercial or financial relationships that could be construed as a potential conflict of interest.
